# Wild boar behaviour during live-trap capture in a corral-style trap: implications for animal welfare

**DOI:** 10.1186/s13028-020-00557-9

**Published:** 2020-11-10

**Authors:** Åsa Fahlman, Johan Lindsjö, Therese Arvén Norling, Petter Kjellander, Erik Olof Ågren, Ulrika Alm Bergvall

**Affiliations:** 1 SLU Swedish Biodiversity Centre, Department of Urban and Rural Development, Swedish University of Agricultural Sciences (SLU), 75007 Uppsala, Sweden; 2 Department of Animal Environment and Health, SLU, 75007 Uppsala, Sweden; 3 Genome Engineering Zebrafish National Facility, SciLifeLab, Uppsala University, 75236 Uppsala, Sweden; 4 Grimsö Wildlife Research Station, Department of Ecology, SLU, 73091 Riddarhyttan, Sweden; 5 Department of Pathology and Wildlife Diseases, National Veterinary Institute (SVA), 75189 Uppsala, Sweden

**Keywords:** Ethogram, Health, Hunting, Management, Refinement, Stress, *Sus scrofa*, 3R, Trapping, Wildlife

## Abstract

**Background:**

Wildlife traps are used in many countries without evaluation of their effect on animal welfare. Trap-capture of wild animals should minimise negative effects on animal welfare, irrespective of whether the animals are trapped for hunting, research, or management purposes. Live-trap capture of wild boar (*Sus scrofa*) followed by killing inside the trap by gunshot is a recently introduced but disputed hunting method in Sweden. Approval of trap constructions is based on gross necropsy findings of 20 trapped and shot wild boars. For improved animal welfare evaluation, our aim was to study wild boar behaviour during live-trapping in a 16 m^2^ square corral-style trap. Behavioural assessments were conducted after filming 12 capture events of in total 38 wild boars (five adults, 20 subadults, 13 piglets). Selected behavioural traits were compared with pathological changes (trap-related lesions) found at necropsy of the 20 subadults, to determine if these variables were useful proxies of capture-induced stress in wild boar.

**Results:**

The wild boars spent less time resting in the evening than in the night and morning. Using Friedman’s ANOVA, there was an overall difference in the time spent foraging. However, we only found a difference between the evening and morning in the Wilcoxon matched pairs test after the Sequential Bonferroni correction, where the wild boars spent more time foraging in the evening than in the morning. Single captured individuals showed more escape behaviours and reacted more strongly to external stimuli than individuals captured in a group. It was more common for animals to charge against the mesh walls of the trap upon human approach compared to upon initial capture when the trap door closed. Trap-related pathological findings due to trauma were documented in 13 of the 20 subadults that were necropsied. Behavioural alterations indicative of capture-induced stress (e.g. charging into the trap walls) were documented in trapped wild boars with no or minor physical injuries (e.g. skin abrasions, subcutaneous haemorrhage).

**Conclusions:**

Behavioural assessment provided valuable information for determination of capture-induced stress in wild boar when evaluating live-trapping in a corral-style trap, whereas pathological evaluation through necropsy did not fully reflect the animal welfare aspects of live-trapping. We emphasize the inclusion of species-specific behavioural data assessment for evaluation of capture-related stress during live-trapping and for testing of new trap constructions before approval.

## Background

The wild boar (*Sus scrofa*) population and its distribution is rapidly increasing in Sweden [1]. Population size management of wild boars in Sweden is predominantly carried out by hunting at bait sites or by driven hunts with hunting dogs. Evaluation of live-trap capture of wild boars followed by killing inside the trap by gunshot was initiated in 2010, before approval as a new hunting method in Sweden [2, 3]. However, trapping wild boar has been criticised and is still a debated topic. From an animal welfare, hunting and research ethics perspective, it is critical to thoroughly assess how the capture process affects the animal. Refinement of wildlife capture methods is essential to minimise stress and improve wild animal welfare, which is in accordance with the principles of the 3Rs—Replacement, Reduction and Refinement [4, 5].

Live-trap capture is not only used for hunting and culling, but also for research and translocation and may have several short- and long-term negative effects on wild animal health and welfare [6–8]. In addition to physical injuries, other stressors such as fear, pain, and poor environmental conditions [9] can all result in a stress response, i.e. behavioural and physiological alterations [10–12]. The impact of stressors on the animals’ welfare is dependent on the animal’s ability to cope with the situation [13, 14]. The effects of live-trap capture on animal welfare while the animals are in the trap are commonly evaluated by only documenting physical injuries or mortality (i.e. conspicuous adverse animal welfare events), while the impact of stress seldom is included [9, 15]. When animals are held in captivity, factors important for welfare include ability to perform natural behaviours, predictability and suddenness of events, control, and familiarity [16]. Several of these factors can be compromised when an animal is captured in a trap. Therefore, changes in activity levels or in behaviours, like foraging and escape attempts, can be used to evaluate animal welfare [17]. For example, an animal that experiences fear or feels threatened may make escape attempts [10, 12]. Thus, the animals’ behaviour is an important component in trap evaluations. In addition, altered post-capture behaviour has been documented in live-trapped wild boar that were released after trapping and anaesthesia [18]. In Sweden, most new live-trap constructions for wild animals require field testing for certification by the Swedish Environmental Protection Agency (SEPA), whereby animal welfare presently is evaluated mainly through pathological examination (gross necropsy findings) of trapped and shot animals [19]. Striving for a more complete evaluation, there is a need to improve the animal welfare assessment of live-trapping by including behavioural evaluations of stress in the protocol. However, there is a lack of scientific studies on wild boar behaviour during live-trap capture.

The overall aim of the study was to evaluate behaviour during live-trap capture of wild boar in a corral-style trap to expand animal welfare assessment during trap testing. We assessed (1) selected behavioural traits, and (2) compared behaviour with pathological changes, to determine if these variables were useful proxies of capture-induced stress in wild boar. We predicted that trapped wild boar would (1) exhibit behaviours indicative of capture-induced stress, and (2) that the behaviours would differ depending on if the animals were captured in a group or as single individuals. Finally, we predicted that (3) stress induced by live-trap capture could be identified by behavioural changes, whereas trap-related physical injuries may be present or not.

## Methods

**Fig. 1:**
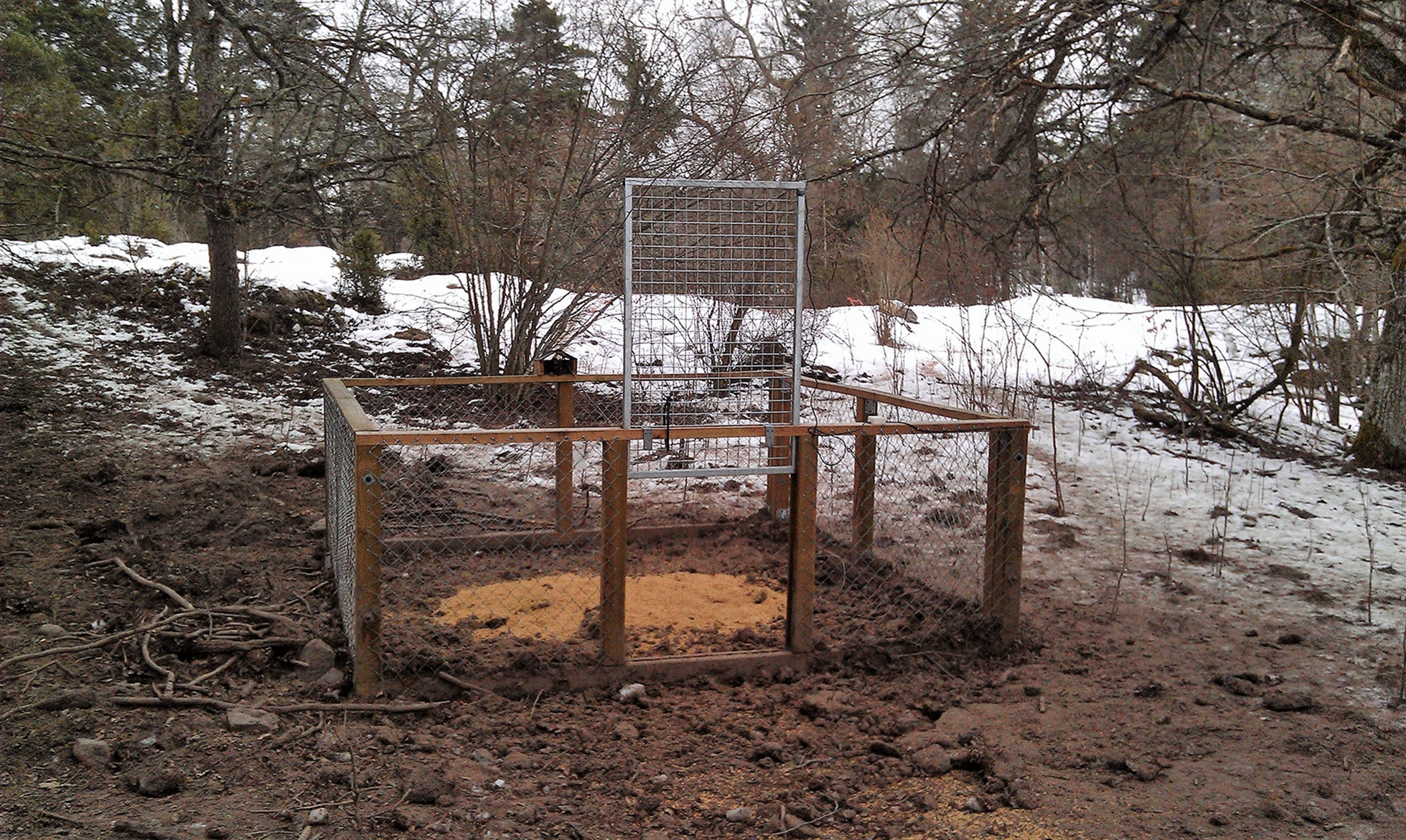
Corral-style trap (JP BUR, https://www.jpbur.se) for live-capture of subadult wild boar in Sweden. This trap was tested based on pathological examinations as part of an assignment from the Swedish Environmental Protection Agency (SEPA) to the Swedish University of Agricultural Sciences (SLU) to evaluate new live-traps for wildlife capture. In the present research study, we analysed behavioural data from films recorded during captures for the trap evaluation

Live-trap capture of free-ranging wild boar in a 16 m^2^ square corral-style trap (Fig. 1) (JP BUR, Oskarström, Sweden) was conducted from 11 March to 21 April, 2015, at Wij Säteri, Bålsta, Sweden (Lat: 59.59, Long: 17.43). The captures were conducted as part of the assignment from SEPA to the Swedish University of Agricultural Sciences (SLU)—Department of Ecology at Grimsö Wildlife Research Station, to evaluate new live-traps for wildlife capture. Approval to test traps by capture of free-ranging wild boar, and subsequent killing of 20 subadults by gunshot, was given by the Ethical Committee on Animal Research, Uppsala, Sweden (Ethical permit C122/13). The animal welfare aspects of the trap evaluation by SEPA was based mainly on pathological examinations through gross necropsy findings. Blood samples were collected post-mortem, but not as a part of the SEPA assignment, and will be presented elsewhere. For this research study, we analysed behavioural data from films recorded during captures for the trap evaluation. The evaluated corral-style trap was approved in 2015 for use in Sweden for live-capture of subadult wild boar during a restricted time of the year; from 1 September until 30 April. Other restrictions included e.g. that the trap door must be set to a maximum opening height of 55 cm to prevent capture of older and thus larger wild boar, and that the trap can be set earliest at 2 h before sunset and must be deactivated at latest 1 h before sunrise.

### Trapping procedure

Ten months before trapping started, the corral-style live-capture trap was placed directly on the ground at the capture site in a forest close to a meadow. The trap construction has a framework of wood studs (trap size, length × width × height; 400 × 400 × 120 cm), with walls of wire mesh with a wire spacing of 4 × 4 cm, and a mesh door of guillotine model (width 100 cm). The mesh door is held open by a pin, which is pulled out when an animal moves a wire trigger inside the trap. Baiting with wheat was initiated sporadically in summer 2014, and regularly in winter and spring 2015 so the wild boar could get used to visiting the trap, before it was set for capture throughout March and April 2015. By distributing the wheat in various amounts and areas inside the trap, it was possible to some extent influence the number of animals that entered the trap before an animal triggered the trap wire that closed the door. At the first capture event, the wheat was finished within 2.5 h; thus, a larger amount of wheat was placed in the trap for all following captures, so feed would last throughout the night, until inspection at dawn. During trapping in March and April 2015, the ambient temperature ranged between 0 and 7 °C, which is within the expected temperature range that time of year.

All captures were conducted by the same experienced wildlife manager. In accordance with the ethical permit, the first five subadult wild boar that were captured were approached and shot after approximately 2.5 h in the trap (Table 1). During the following captures, the trap was approached the morning after capture, when the wild boar had spent 11–14 h in the trap (Table 1).

**Table 1:** Data from 12 capture events of 38 wild boars (five adults, 20 subadults, 13 piglets) for the evaluation of a corral-style trap

Capture event	Number of captured animals	Time when the trap was set	Time of capture	Time when manager arrives	Total time in trap
1	4	17:42	18:48	21:17	2 h 34 min
2	1	17:50	19:37	22:04	2 h 27 min
3	3	17:18	20:44	08:20	11 h 22 min
4	2	17:21	18:24	08:30	13 h 56 min
5	1	17:30	18:38	08:30	13 h 52 min
6	4	18:12	19:44	08:05	12 h 23 min
7	11^a^	18:48	20:22	09:01	12 h 37 min
8	2^b^	18:30	19:33	07:35	11 h 5 min
9	6^c^	18:05	20:40	08:47	12 h 7 min
10	1	19:34	19:47	07:48	11 h 10 min
11	1	19:24	20:29	08:30	12 h 4 min
12	2	20:04	20:24	08:24	10 h 57 min

^a^One sow and 10 piglets. The sow jumped out of the trap at 07:55 and the piglets were released

^b^Two wild boars were initially captured, but one jumped out of the trap at 05:28

^c^Three sows and three piglets were captured. Upon trap approach by the wildlife manager, the animals got stressed inside the trap and one piglet was trampled, severely injured and euthanised on the spot, whereas the other five animals were released

In total, 38 wild boars of different age groups were captured during 12 different capture events (Table 1). We defined piglets as individuals with striped fur (approximately 0–5 months), subadults as individuals with reddish or brownish fur without stripes (approximately 5–10 months), and adults based on dentition and reproductive tract. Of the 38 captured wild boar, two of five adults jumped out of the trap, 16 animals were released (three sows and 13 piglets) as these animals were not the target animals for trapping, whereas 20 subadults were shot for pathological assessment within the SEPA assignment. A total of 20 subadults were required to be captured and necropsied for the trap evaluation. The subadults were killed by gunshot to the brain (0.22 LR cartridge used in a revolver or a rifle) by the wildlife manager that conducted all captures. When piglets were captured, all animals in the trap were released (Table 2), except on one occasion when one piglet was severely injured and had to be euthanised. There was no follow-up of the released wild boar post-capture. Single individuals were captured in the trap during four of the 12 capture events (Table 1). During eight capture events, more than one wild boar (two to 11 individuals) were captured at the same time.

**Table 2:** Ethogram used for the evaluation of live-trap capture of wild boar in a corral-style trap

Behavioural categories	Behaviour of focal individual	Behavioural descriptions
Rest	Resting	Lying down, usually together with others. The behaviour is preceded by bedding behaviour performed in a calm way
Still	Standing still	Standing without any foraging attempts or taking maximum two steps
Forage	Foraging	Foraging behaviour including searching, rooting, eating and scraping with forelegs
Active	Walking	Walking with short pauses, exploring the environment within the trap, or interacting with other individuals in an exploratory or neutral way
	Moving fast	Moving fast and pauses between the fast movements are less than five seconds
	Chasing	Chasing or being chased by another individual
	Biting	Biting or being bitten by another individual
Escape	Biting mesh wall	Biting the mesh wall
	Rearing against wall	Rearing up on its back legs and putting the front legs on the wall or the door
	Charging into wall	Charging into the mesh wall or door with the snout or other body parts

### Behavioural observations

A video camera (EYE-02, Jablotron Alarms a.s., Jablonec nad Nisou, Czech Republic) with infra-red light (IR LED reflector 0.6 W, 850 nm, angle 80°, Resolution 640 × 480 AVI video) was placed at a height of 3 m on a pole 1 m from the trap to record the whole trap, its entrance and the nearby surroundings. The camera was powered by a 12 V car battery and had an infra-red motion detector that detected temperature changes in front of the camera, and a motion-in-picture detector. After 15 min (min) of inactivity, the camera stopped recording, and resumed recording upon new movements. An ethogram was established, and the behaviours were divided in five categories (Table 2). The behavioural observations were conducted by two observers (TAN and UAB). Behavioural data were collected by observing subadult and adult wild boars using continuous monitoring of focal individuals, where the researcher observed and recorded the behaviour of one individual at a time [20]. Due to interrupted filming when all animals in the trap were inactive, it was not possible to observe one specific individual continuously. Thus, different focal individuals were observed to reflect the average group behaviour. For the first five wild boars, we analysed filmed behaviours for approximately 2.5 h, from capture until the animals were shot. For the remaining wild boars, behavioural observations were conducted on three separate time periods of each capture event (up to 5 h film per capture); evening (from capture and 2 h ahead, before 23:00), night (1 h between 23:00–05:00) and morning (2 h after 5:00 until the animals were shot). In total, we observed 43.5 h of film from 12 different captures. The total number of charges into the trap walls or door was counted upon initial capture and upon approach by the wildlife manager for individual subadults and adults (not focal individuals for this behaviour), divided by the number of individuals per capture. The total time from arrival of the wildlife manager until all trapped wild boars were shot was recorded from the films (Table 1).

### Necropsies

Pathological assessment of trap-related lesions found at necropsy of 20 subadult wild boars was conducted as part of the assignment from SEPA, and we used the gross necropsy findings for comparison with our research data on behaviour of captured adult and subadult wild boar. Necropsies were conducted by wildlife pathologists at the Department of Pathology and Wildlife Diseases at the National Veterinary Institute (SVA), Uppsala, Sweden. Necropsies were conducted the same day as the animals were shot, except for the five first animals which were shot in the evening and necropsied the following day.

Any lesions noted at necropsy by the veterinary pathologists were standardised by one author (EÅ). The necropsies followed the standard procedure of SVA, with a focus on tissues and organs that could be expected to sustain lesions due to the capture period; skin, subcutaneous tissue, face including snout, eyes, and ears, mouth including lips, teeth, gingiva, and tongue, extremities including claws, tendons, ligaments, and skeleton. With regard to internal organs, the focus was on the stomach and its mucosa to assess if potential acute stress-related erosions or haemorrhage had developed. All internal organs were inspected and sectioned or opened. For any lesion found at necropsy, an evaluation of age of the lesion was done. Acute lesions suggestive of being acquired at capture or within the trap were noted as trap-related. The lesions were scored following the NFS 2013:13 appendix 2, a modified version originating from the ISO standards [15]. Subacute and chronic, apparently older lesions were noted separately.

### Statistical analyses

We used non-parametric tests for all comparisons. When two or more individuals were captured, different focal individuals were observed to represent the average group behaviour, thus the value included in the behavioural analyses represents the group. When we tested the difference between the proportions of performed behaviours at different points in time (evening, night and morning), we first analysed the data with Friedman’s ANOVA. When we found a difference, we performed a pairwise test, the Wilcoxon matched pairs test. We corrected for multiple analysis with the Sequential Bonferroni correction [21]. The Wilcoxon matched pairs test was used to compare the number of charges against the mesh wall per individual upon initial capture in the trap and upon human approach. The Mann–Whitney U test was used to compare single individual versus group captures. All analyses were performed in Statistica 13 (Statsoft) and the significance level was set to *P* < 0.05.

## Results

The median time from when the trap was set until wild boars were captured was 85 min (range 62–206 min). No non-target species were captured. The median time from arrival of the wildlife manager until all wild boars were shot was 5.4 min for group captures (range 1.6–11.1 min) and 1.0 min for single captures (range 0.7–1.6 min).

### Behavioural observations

**Fig. 2:**
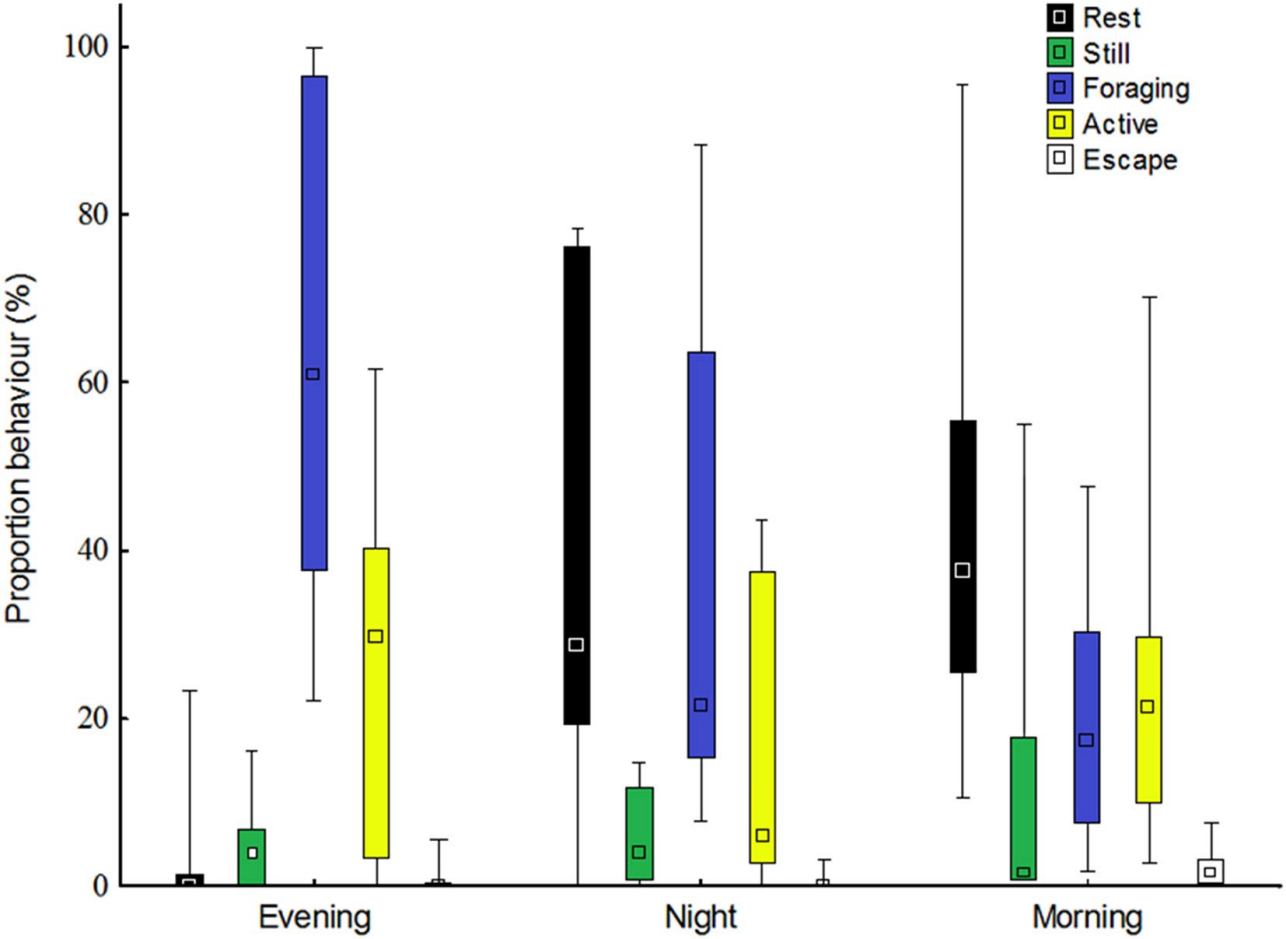
Distribution of behaviours of wild boar captured in a corral-style trap. Nine capture events with data from the evening, night and morning were included in the analysis. The median (square), maximum and minimum (whiskers) and the 25–75% quartiles (box) are shown

We found that the wild boar spent less time resting in the evening than in the night and morning (Fig. 2, Tables 3 and 4). Using Friedman’s ANOVA, there was an overall difference in the time spent foraging (Fig. 2, Table 3). However, we only found a difference between the evening and morning in the Wilcoxon matched pairs test after the Sequential Bonferroni correction, where the wild boars spent more time foraging in the evening than in the morning (Fig. 2, Table 4). There was no difference in the proportion of time that the animals were active, still or performed escape behaviours comparing the evening, night and morning (Tables 3 and 4). Additional file 1 shows filmed behaviour of a single captured male wild boar that was foraging, and Additional file 2 shows capture of four subadult wild boars that were active and foraging, while three other wild boars were walking around outside the trap.

**Table 3:** The median time (%) wild boars spent performing different behaviours in a corral-style trap

Behavioural category	Median time (%) spent on each behaviour^a^			*χ*^2^	df	*P*
	Evening	Night	Morning			
Rest	0	29	38	8.7	2	0.013*
Still	4	4	2	0.5	2	0.77
Forage	61	21	17	9.6	2	0.008*
Active	30	6	21	2.8	2	0.25
Escape	0	0	2	7.5	2	0.023*^,b^

^a^To test the difference between the behaviour performed during the evening, night and morning, a Friedman ANOVA was performed

^b^The significant difference disappeared in the pair-wise test (Wilcoxon matched pairs test) after the Sequential Bonferroni correction

*Significant difference was set to *P* < 0.05

**Table 4:** Behavioural comparison from nine capture events of wild boars in a corral-style trap

Comparison^a^	Rest		Forage		Escape	
	T	P	T	P	T	P
Evening and night	0	0.036*	8	0.17	9	0.40
Evening and morning	1	0.033*	0	0.023*	3	0.11
Night and morning	20	0.77	15	0.37	5	0.14

*Significant difference was set to *P* < 0.05

^a^Results from the Wilcoxon matched pairs test for the behaviours rest, forage and escape

**Fig. 3:**
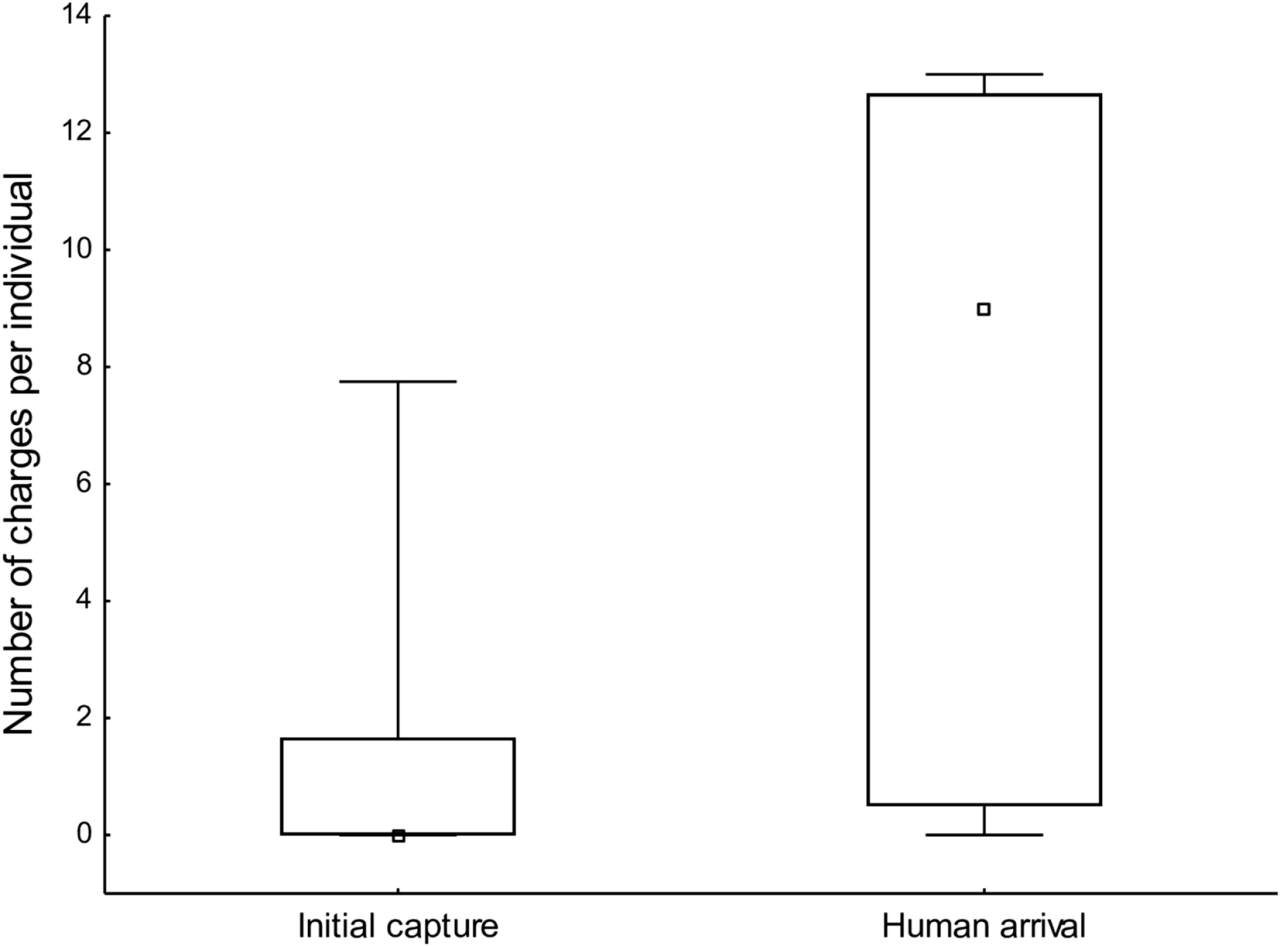
Number of charges by captured wild boar into the mesh wall or door of a corral-style trap. Charges upon initial capture (11 capture events) and upon approach by the wildlife manager (7 capture events) are shown as the median (square), maximum and minimum (whiskers) and the 25–75% quartiles (box)

**Fig. 4:**
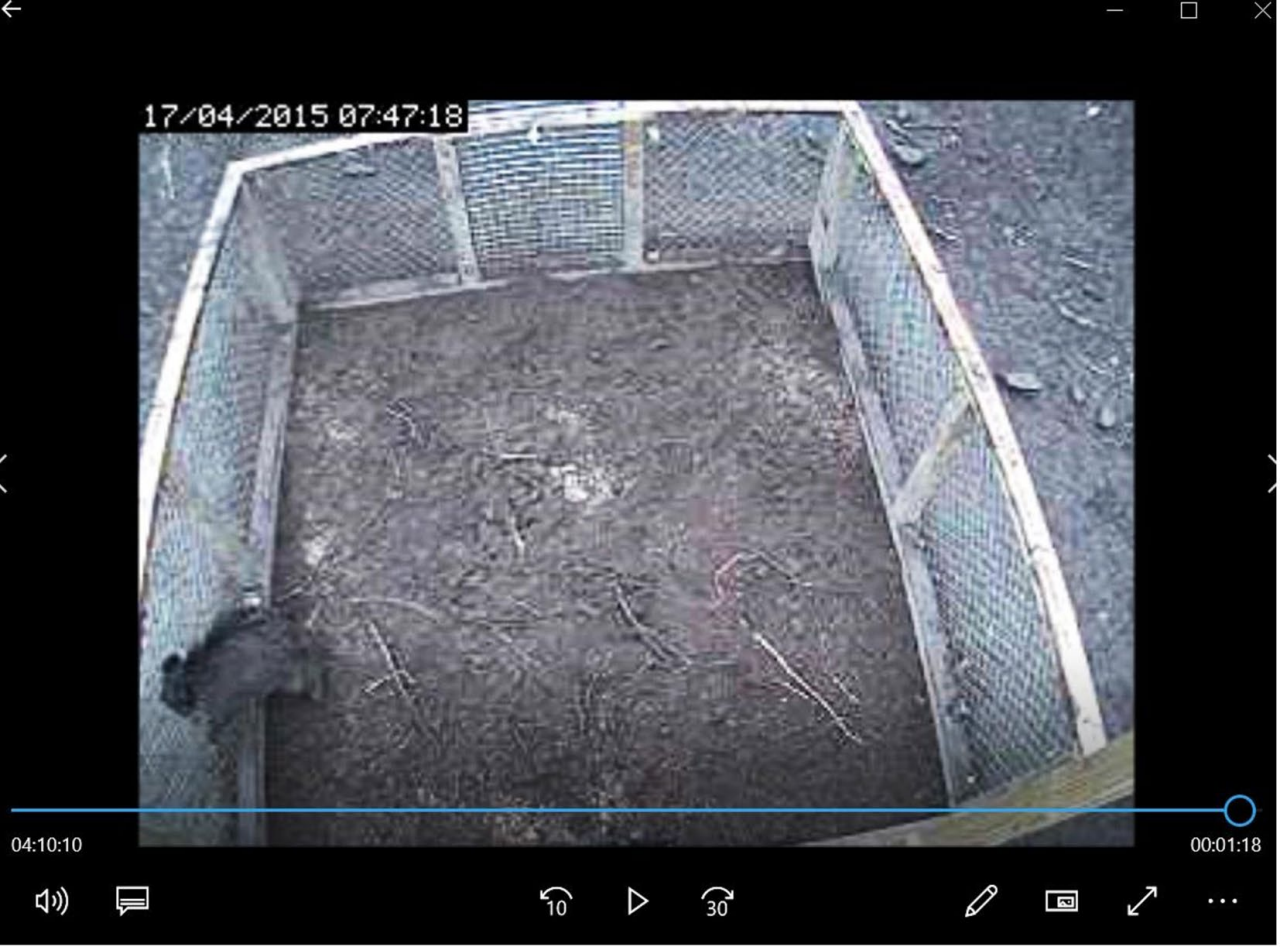
Wild boar charging against the mesh walls of the corral-style trap

Upon initial capture, individuals charged into the mesh walls when the trap door closed in 4 of 11 captures, whereas individuals charged into the mesh walls or door when the wildlife manager approached the trap at six of seven filmed approaches. It was more common for animals to charge against the mesh walls of the trap upon human approach compared to upon initial capture when the trap door closed (Wilcoxon matched pair test; n = 7, T = 1, *P* = 0.028, Figs. 3 and 4). Some of the behaviours differed in terms of whether the animals were captured in a group or as single animals. Single wild boar reared against the wall more frequently compared to animals captured in a group of two or more individuals (Mann–Whitney *U* test; Z = -2.29, *P* = 0.022, n_Single_ = 4, n_Group_ = 8). We found no difference in time spent resting between single wild boar and animals captured in a group (Mann–Whitney U test; Z = 0.08, *P* = 0.93, n_Single_ = 4, n_Group_ = 8). When the focal individual within a group was resting, 95.5% of the time all animals in the group were resting. Single-captured wild boar reacted with stronger startle response to external stimuli than animals captured in a group.

### Pathological assessment

Trap-related pathological findings were documented in 13 of the 20 subadults (Table 5). Minor and superficial acute skin abrasions on the snout, nasal bridge, or chin, with minor localized areas of subcutaneous haemorrhage were documented in 12 of 20 animals (lesion score 5, Table 5). One wild boar had an acute lesion with moderate local tissue damage and a 40 mm long fissure in the nasal bone with abrasions on the overlying skin and acute subcutaneous haemorrhage (lesion score 30, Table 5).

**Table 5:** Trap-related pathological findings documented in 13 of 20 subadult

Capture event	Sex^a^	Body mass (Kg)	Superficial skin abrasions; location and size (mm)	Subcutaneous haemorrhage; location and size (mm)	Bone injuries, size (mm)	Lesion score^b^
3	F	23	–	Nasal bridge, 20 × 25	–	5
3	F	23	–	Nasal bridge, 20 × 25	–	5
3	F	24	–	Nasal bridge, 20 × 25	–	5
5	M	42	Snout, 10 × 5	Nasal bridge, 20 × 25	–	5
6	M	21	–	Nasal bridge, 20 × 25	–	5
6	M	37	Snout × 2, nasal bridge, 30 × 35, 7 × 7, 25 × 14	Nasal bridge, 20 × 25	Nasal bone fissure, 40	30
6	F	33	–	Nasal bridge, 20 × 25	–	5
6	F	36	–	Nasal bridge, 5 × 5	–	5
8	M	28	Snout × 2: 15 × 10, 6 × 6	Nasal bridge × 2: 55 × 40, 30 × 15	–	5
10	F	26	Snout, 15 × 10	Chin, 30 × 15	–	5
11	M	50	Nasal bridge, 20 × 15	Nasal bridge, chin 20 × 15, 5 × 5	–	5
12	M	40	Nasal bridge × 2: 15 × 6, 5 × 5	Nasal bridge, 50 × 20	–	5
12	M	47	Snout × 2, nasal bridge, 30 × 10, 8 × 10, 8 × 8	Nasal bridge, 50 × 30	–	5

Trap-related pathological findings were documented in 13 of 20 subadult wild boars that were captured in a corral-style trap and killed by gunshot to the brain

^a^Sex: F-female, M-male

^b^Lesion score according to the Swedish Environmental Protection Agency’s Regulations on approval of Hunting Devices, (*In Swedish*) Naturvårdsverkets föreskrifter om typgodkännande av fångstredskap, NFS 2013:13, Naturvårdsverkets Författningssamling

Available at: https://www.naturvardsverket.se/Documents/foreskrifter/nfs2013/nfs-2013-13.pdf

## Discussion

We documented behavioural alterations indicative of capture-induced stress in animals with no or minor physical injuries; thus, our assessment provided a deeper understanding of the animal welfare in trap-captured wild boars. Trap-related injuries did not fully reflect capture-induced stress, whereas behavioural alterations added further information on the extent of the stress response. In line with our prediction, stress behaviour varied between animals captured alone or in a group.

Wildlife traps are used in many countries with no evaluation of animal welfare. Behavioural or physiological measures of animals captured in different trap types are rarely reported in scientific studies [9]. The corral-style trap in the present study was approved for wild boar capture in Sweden after assessment that was based on scoring of physical injuries. In animals with no or minor lesions, behavioural observations revealed a high stress response that was not reflected by the physical injuries acquired in the trap. External threats perceived by an animal during live-trapping affect its behaviour and may generate negative effects such as anxiety, fear or panic, in line with the Five Domains for animal welfare assessment, as described in Mellor and Beausoleil [22].

In the present study, wild boar captured in the corral-style trap performed similar behaviours as earlier documented in free-ranging wild boars [23], but because of capture, the study animals also performed escape- and exploratory behaviours. Foraging and resting are indirect measures of stress, i.e., a stressed individual performs less of these behaviours [24]. Our findings suggest that the ability to rest in the trap is important for welfare. Other studies have found that shade and mud are preferred for sleeping and resting [23] and soil is a good substrate for rooting, especially if there is food to find. In Sweden, some traps approved for wild boar capture have a metal or wooden floor [3], which limits the possibility to perform natural behaviours as foraging and resting comfortably. In addition, traps for single animal capture will lead to social isolation. In our study, the behaviours reflecting stress differed when a single individual was captured in the trap, compared to when a group was captured. We found that single captured individuals showed more escape behaviours and reacted more strongly to external stimuli than individuals captured in a group. This finding corroborates the idea that isolation itself can elicit stress, especially in animals that live in groups, such as pigs [25, 26]. Mesh walls might prevent single captured individuals from social isolation to some extent when groups of wild boars are foraging outside the trap. On the other hand, animals get injured when charging the mesh walls. The frequency of traumatic injuries and mortalities have been reported in wild boars captured in different corral-style traps [27–30]. Gaskamp [28] reported that every wild boar capture in corral traps resulted in animals running and jumping into all sides of the trap. Sweitzer et al. [30] described that the first six wild boars that were captured in a square steel mesh panel trap suffered injuries from lacerations and abrasions to avulsed lower lips and fractured nasal bones from charging the steel mesh panels during escape attempts. The frequency and severity of trap-related injuries decreased after the trap was modified by adding a net on the inside to prevent contact with the panels, and a runway leading to an expanded corral section [30]. No gross pathology was documented in wild boars captured in three different types of wire net traps with different sizes that were covered with wood panels or branches on the inside [29]. Mortalities related to severe trauma and associated euthanasia have been reported in wild boar captured in corral traps [27]. In our study, trap-related injuries mostly occurred on the snout and nasal bridge, which likely are compatible with the wild boar charging against the mesh wall, as documented upon initial capture and when the wildlife manager approached the trap to euthanise the animals. Human presence most likely elicited the highest stress response during capture and thus risk of injuries since it was more common for wild boars to charge against the mesh wall upon human approach. Potentially, solid walls may reduce this risk by blocking the animal’s vision of an approaching person, but solid walls will also increase the isolation of a single trapped wild boar and may affect the animal’s willingness to enter the trap. A stress reaction triggered by the presence of humans has been shown during capture of roe deer (*Capreolus capreolus*) in box traps [31]. The high likelihood for animals to become injured at human arrival should be considered during trap construction and evaluations. In addition, when the wild boar were running around or charging against the mesh wall upon human approach, it was more difficult to immediately kill the animals by gunshot to the brain within this corral-style trap (Petter Foucard, pers. communication).

Although traps intended for groups might be less stressful for some individuals, they can be harmful in some situations. One example is when a sow is separated from all or some of her piglets, as documented in the present study. Piglets have poor thermoregulation and need the sow for food and to keep warm [32]. In addition, if more than one adult is caught in the trap together with piglets, the restricted space increases the risk of injuries to piglets. In the present study, one of the 13 piglets was severely injured and had to be euthanised. In Sweden, the trap door opening of traps approved for live-capture of wild boars must be maximum 55 cm in height to prevent sows from entering the trap, otherwise they would be unable to feed dependent piglets that remain outside the trap. If only piglets go inside the trap, the trap door will not close since piglets are too small to trigger it. This study and evaluations of similar trap constructions have shown that the height limitation does not always stop adult wild boar from entering (Erik Ågren, pers. communication). The trap door of the evaluated trap is one metre wide, but the maximum width is not regulated; a size limitation of the width of the entrance would probably be needed to exclude larger adult animals. In conclusion, each trap type has its limitations, and to fully evaluate the welfare consequences of different trap constructions several aspects of the trapping need to be considered. A limitation of the behavioural data collection in this study was that the filming was interrupted when all individuals in the trap were inactive, which made it impossible to separate individuals in a group throughout the capture events. Thus, the methodology of studying different focal individuals was chosen to reflect the average group behaviour.

Our results imply that evaluating only pathological findings, such as injuries, does not fully reflect animal welfare during wild boar trapping. Similarly, Marks [33] emphasises that injuries or death in restraining traps are only end-points of poor trapping welfare. Iossa et al. [9] suggested already in 2007 that evaluation of trapping effects on captured animals should include pathological as well as behavioural and physiological assessment. In addition, the ‘*Agreement on international humane trapping standards between the European Community, Canada and the Russian Federation’* stated in 1998 that each member state should support research to improve existing trap-testing protocols, including development and validation of physiological and behavioural test protocols for evaluation of animal welfare [34]. For a more complete evaluation, there is a need to improve animal welfare assessment of live-trapping by including evidence-based behavioural and physiological evaluations of stress in the protocol. In the present study, blood samples were collected post-mortem for analysis of new biomarkers of stress, which will be presented elsewhere. Further work is needed to establish a validated repeatable animal welfare assessment protocol through behavioural studies for wild boar and other species when using different capture methods. Also, further studies on physiological variables during capture is warranted. Long-term effects post-capture and survival rates for live-trapped animals that are released should be studied.

## Conclusions

We found that capture-induced stress in the wild boar, documented by behavioural alterations, may result in no or only minor physical injuries. Thus, capture-related injuries alone did not fully reflect the level of stress induced by live-trap capture. The corral-style trap was approved for live-capture of wild boar according to present regulations. These are based mainly on pathological evaluation of trapped and killed animals, which alone does not capture all animal welfare aspects. We therefore emphasize the need for the inclusion of behavioural data assessment for the evaluation of capture-related stress in wild boar during live-trapping and for testing of new trap constructions before approval.

## Supplementary information

**Additional file 1.** Foraging behaviour of a subadult male wild boar captured in a corral-style trap. The film shows how a single captured wild boar (capture event #5) was active and walked with short pauses, exploring the environment within the trap (JP-BUR). The wild boar was rooting in the ground and was eating the wheat that the trap was baited with; this behaviour was categorised as forage in the ethogram for evaluation of live-trap capture of wild boar.

**Additional file 2.** Capture of four subadult wild boars in a corral-style trap. The films shows how four wild boars got captured (capture event #6) in a corral-style trap (JP-BUR). Three other wild boars were moving around outside the trap. The trapped wild boars were rooting in the ground and eating the wheat that the trap was baited with; the behaviour was categorised as forage.

## Data Availability

The datasets used and/or analysed during the current study are available from the corresponding author on reasonable request.
